# Effects of Filler Functionalization on Filler-Embedded Natural Rubber/Ethylene-Propylene-Diene Monomer Composites

**DOI:** 10.3390/polym14173502

**Published:** 2022-08-26

**Authors:** Sung-Hun Lee, Gun-Woo Park, Hee-Jun Kim, Kyungho Chung, Keon-Soo Jang

**Affiliations:** Department of Polymer Engineering, School of Chemical and Materials Engineering, The University of Suwon, Hwaseong 18323, Gyeonggi-do, Korea

**Keywords:** phlogopite, natural rubber (NR), ethylene-propylene-diene (EPDM), rubber composite, compatibilizer, coupling agent, mechanical properties

## Abstract

Natural rubber (NR) presents a number of advantages over other types of rubber but has poor resistance to chemicals and aging. The incorporation of ethylene propylene diene monomer (EPDM) into the NR matrix may be able to address this issue. Mineral fillers, such as carbon black (CB) and silica are routinely incorporated into various elastomers owing to their low cost, enhanced processability, good functionality, and high resistance to chemicals and aging. Other fillers have been examined as potential alternatives to CB and silica. In this study, phlogopite was surface-modified using 10 phr of compatibilizers, such as aminopropyltriethoxysilane (A1S), aminoethylaminopropyltrimethoxysilane (A2S), or 3-glycidoxypropyltrimethoxysilane (ES), and mixed with NR/EPDM blends. The effects of untreated and surface-treated phlogopite on the mechanical properties of the rubber blend were then compared with those of common fillers (CB and silica) for rubbers. The incorporation of surface-modified phlogopite into NR/EPDM considerably enhanced various properties. The functionalization of the phlogopite surface using silane-based matters (amino- and epoxide-functionalized) led to excellent compatibility between the rubber matrix and phlogopite, thereby improving diverse properties of the elastomeric composites, with effects analogous to those of CB. The tensile strength and elongation at break of the phlogopite-embedded NR/EPDM composite were lower than those of the CB-incorporated NR/EPDM composite by 30% and 10%, respectively. Among the prepared samples, the ES-functionalized phlogopite showed the best compatibility with the rubber matrix, exhibiting a tensile strength and modulus of composites that were 35% and 18% higher, respectively, compared with those of the untreated phlogopite-incorporated NR/EPDM composite. The ES-functionalized phlogopite/NR/EPDM showed similar strength and higher modulus (by 18%) to the CB/NR/EPDM rubber composite, despite slightly lower elongation at break and toughness. The results of rebound resilience and compression set tests indicated that the elasticity of the surface-modified phlogopite/NR/EPDM rubber composite was higher than that of the silica- and CB-reinforced composites. These improvements could be attributed to enhancements in the physical and chemical interactions among the rubber matrix, stearic acid, and functionalized (compatibilized) phlogopite. Therefore, the functionalized phlogopite can be utilized in a wide range of applications for rubber compounding.

## 1. Introduction

Rubbers feature various properties, including elasticity and ductility. These materials are typically blended with different materials, including other types of rubber, because using singular rubber is unable to show multi-functional properties simultaneously [1,2]. Natural rubber (NR) has been widely used in a myriad of applications, such as tires, hoses, gloves, shoes, conveying belts, balls, and cushions, owing to its good strength, wear resistance, resilience (elasticity), and processing characteristics [3,4,5,6,7,8]. However, NR has highly unsaturated backbones, which lead to poor weathering resistances (e.g., to UV, oxygen, ozone, and heat), thereby impeding its outdoor applications [9,10,11,12].

Ethylene-propylene-diene monomer (EPDM) rubber features high mechanical properties and broad operation temperature, thereby enhancing the mechanics, impact strength, and low-temperature stability of polymer composites when incorporated together [13,14,15,16]. EPDM is a highly saturated elastomer comprising a small concentration of nonconjugated dienes; thus, it exhibits good anti-aging properties and resistance to weathering, oxidation, and chemicals [17,18,19]. Polymers, such as polyamide, polyolefin, and wood polymers, can be toughened by incorporation of EPDM and functionalized EPDM [13,15,20]. Mixing EPDM with NR can mitigate the poor weathering resistance of the latter without remarkably affecting its inherent properties of NR [1,19]. Moreover, NR/EPDM blends and composites are used in the applications of tires and brake lining [21,22]. However, most rubber blends are immiscible or only partially compatible owing to phase separation (viscosity mismatch), poor interfacial interactions among the different phases, differences in cure rate (unsaturation degree), polarity, and solubility of additives/curing agents, all of which result in poor mechanical properties [23,24,25,26]. Several investigations have been conducted on the correlations among the rheological (processing), morphological, physical, and mechanical properties of incompatible NR/EPDM rubber blends [1,2,19]. For instance, previous studies revealed that the curing compatibilities of these blends could be enhanced by chemical modifications including the reactive blending of EPDM (halogenation, mercapto- maleic anhydride-, accelerator-, epoxide-grafting, etc.) [1,2,27] and use of accelerators (with elevated solubility for EPDM, longer alkyl chains, and mercapto moieties (sulfenamide and thiuram groups)) [28,29]. Moreover, various compatibilizers, such as methacrylate-butadiene-styrene (MBS), aminosilanes, trans-polyoctylene rubber (TOR), have been also developed [2]. Lastly, EPDM could be precured prior to NR mixing to improve their compatibility [1,30]. NR/EPDM blends are initially vulcanized by consuming the vinyl moieties of NR, whose vulcanization rate is higher than that of EPDM owing to its higher degree of unsaturation [31].

The incorporation of inorganic fillers into rubber blends enhances not only the thermal and mechanical properties but also their interfacial interactions by stabilizing their interfaces. Carbon black (CB), silica, CaCO_3_, clay, zeolite, and organoclay are some examples of multifunctional inorganic fillers [14,32,33] and serve as inorganic compatibilizers. Among these fillers, CB and silica are the most extensively exploited owing to strong interactions between the rubber and filler phases [34,35,36,37]. However, they are somewhat expensive, and the black color of CB hinders a wide range of applications. Thus, potentially used fillers, such as organo-montmorillonite, mica, bio-based fibers, and biochar have been examined as alternatives to these materials [31,38,39]. For instance, mica-based fillers have recently been used not only in a number of general applications (e.g., plastic parts, adhesives, etc.) but also in elastomers [31,40,41].

Phlogopite, a mica derivative, has been used in polymer composites owing to good electrical, thermal, and dimensional stabilities [42,43,44,45]. In this study, phlogopite was functionalized with amino or epoxide groups and utilized to improve the thermal and mechanical properties of NR/EPDM blends. The effects of untreated and surface-treated phlogopite on the mechanical properties of NR blends were then compared with those of common fillers (i.e., CB and silica).

## 2. Experimental

### 2.1. Materials

**Figure 1:**
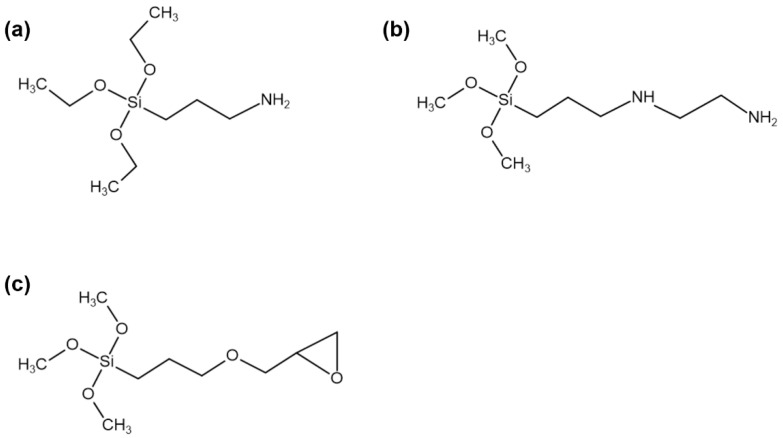
Silane structures: (**a**) aminopropyltriethoxysilane (A1S), (**b**) aminoethylaminopropyltrimethoxysilane (A2S) and (**c**) 3-glycidoxypropyltrimethoxysilane (ES).

Ethylene propylene diene monomer (EPDM, Keltan KEP-330) and natural rubber (NR, STR 5L) were purchased from Kumho Polychem Co. (Seoul, Korea) and PAN STAR Co. (Bangkok, Thailand), respectively. Carbon black (CB; 28–36 nm, N330, Aditya Birla Chemicals Co., Andhra Pradesh, India), silica (20–30 μm, 3M Co., St. Paul, MN, USA), and phlogopite (40–80 μm, LKAM Minerals Co., Luleå, Sweden) were used as particulate reinforcements. Stearic acid (SA), zinc oxide (ZnO), sulfur, *N*-cyclohexyl-2-benzothiazyl sulfonamide (CBS), and tetramethylthiuram disulfide (TMTD) were purchased from Puyang Willing Chemical Co. (Puyang, China). Aminopropyltriethoxysilane (A1S, OFS-6011) and aminoethylaminopropyltrimethoxysilane (A2S, OFS-6020) were supplied by Dow Chemical Co., Midland, MI, USA). 3-Glycidoxypropyltrimethoxysilane (ES), acetic acid, and DI water were purchased from BNOChem Co. (Cheongju, Korea). Three types of silanes are shown in Figure 1.

### 2.2. Rubber Compounding

The mastication (pre-mixing) of NR (75 wt%) and EPDM (25 wt%) was performed using an open two-roll mill until a blend band form (completely blended mixture) was fabricated. Each filler (10 parts per hundred resin (phr)), 5 phr of ZnO, and 2 phr of SA were incorporated into the rubber blend and compounded together for 15 min; the resulting compound was denoted “carbon master batch (CMB)”. Then, 1 phr of TMTD, 1 phr of CBS, and 1 phr of sulfur were added to the mixture and mixed for 5 min; the resulting blend was denoted “final master batch (FMB)”. Recipes of the rubber compounds are listed in Table 1.

### 2.3. Surface Modification of Phlogopite Using Silane-Based Coupling Agents

Phlogopite was surface-modified with amine or epoxide groups using silane-based coupling agents prior to its incorporation into the rubber blend. The pH of the DI water was set to 3.0 using acetic acid. Two phr of each silane-based coupling agent was added to the modification solution (400 mL), which was subsequently stirred at 300 rpm for 15 min. Thereafter, 200 g of phlogopite was added to the solution, which was stirred once more at 300 rpm for 1 h. The resulting solution was oven-dried at 140 °C for 24 h.

### 2.4. Curing Characteristics

#### 2.4.1. Cure Time (T_90_)

The time required for torque to reach 90% of maximum torque during curing (T_90_) was determined at 170 °C using a rubber rheometer (DRM-100, Daekyung Engineering Co., Ulsan, Korea). The mean of five measurements was calculated for each sample.

#### 2.4.2. Mooney Viscosity

A Mooney viscometer (DWV-200C, Daekyung Engineering Co., Ulsan, Korea) was utilized to measure the Mooney viscosity of rubber samples at 125 °C for 4 min. The sample was pre-heated at 125 °C for 1 min to stabilize the sample prior to measurements.

### 2.5. Mechanical Properties

#### 2.5.1. Tensile Properties

Uniaxial tensile deformation was carried out according to ISO 37 using a universal testing machine (UTM; DUT-500CM, Daekyung Engineering Co., Ulsan, Korea). The cross-section of the specimen was 15 mm × 4 mm and the gauge length was 40 mm. The specimens were elongated at a crosshead speed of 500 mm/min with a load cell of 5 kN at room temperature. The mean values were calculated based on five specimens.

#### 2.5.2. Hardness

A hardness tester (306 L, Pacific Transducer instruments, Los Angeles, CA, USA) was utilized to measure the Shore A hardness of the rubber blends and composites according to ISO 48. The mean values were determined based on five specimens.

#### 2.5.3. Abrasion Resistance

An abrasion resistance test was conducted using an abrasion tester. A 2.5 cm × 2.5 cm sample was rotated 200 times at 40 rpm in a tester with a diameter of 15 cm. The abrasion resistance index (ARI) was calculated based on Equation (1):

(1) $$\mathrm{ARI}=\frac{\Delta m_{r}\;p_{t}}{\Delta m_{t}\;p_{r}}\times 100$$

where Δ*m_r_*, *p*_t_, Δ*m_t_*, and *p_r_* represent the mass loss of the reference compound, density of the test rubber, mass loss of the test rubber, and density of the reference compound, respectively.

### 2.6. Elastic Properties

#### 2.6.1. Rebound Resilience

The rebound elasticity of the rubber blends and composites was measured according to ISO 4662 using a ball rebound tester (H090, UTS International Co., Zhangzhou, China). The specimens were maintained at room temperature for 2 h prior to the measurements. A ball was dropped onto the samples, and its rebound height was measured. The mean of five measurements was determined for each sample.

#### 2.6.2. Compression Set

Compression set tests were performed according to ISO 815. The specimens were placed into a cylindrical mold with dimensions of 12.5 mm (±0.5 mm) × 9 mm and compressed at 125 °C for 24 h. Subsequently, the compressed specimen was removed and allowed to rest until it regained its form. The final dimensions (i.e., height) of the specimens were measured and the mean value was determined based on the four specimens.

### 2.7. Morphology

The morphologies of the NR/EPDM blend and NR/EPDM/phlogopite composites were observed using a scanning electron microscope (SEM; Apro, FEI Co., Hillsboro, OR, USA) at an electron beam voltage of 10.0 kV. The surface fractured during tensile tests was coated with a 5–10 nm-thick gold layer using a sputter coater (Cressington 108 Auto Sputter Coater, Ted Pella Inc., Redding, CA, USA) prior to the SEM measurements.

## 3. Results and Discussion

**Figure 2:**
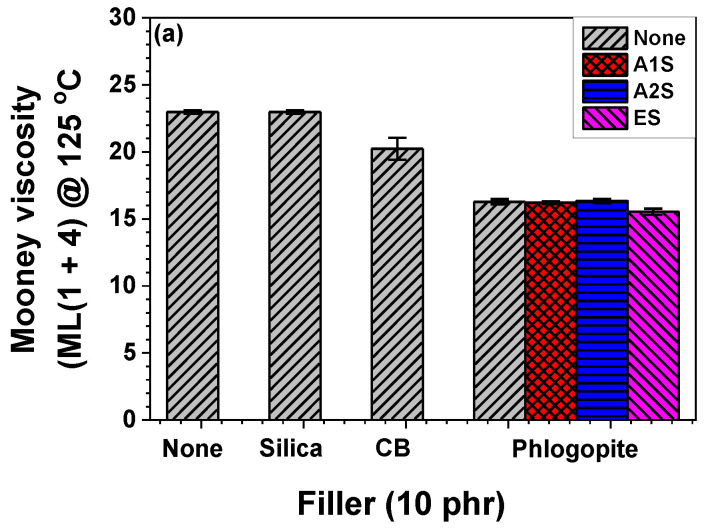

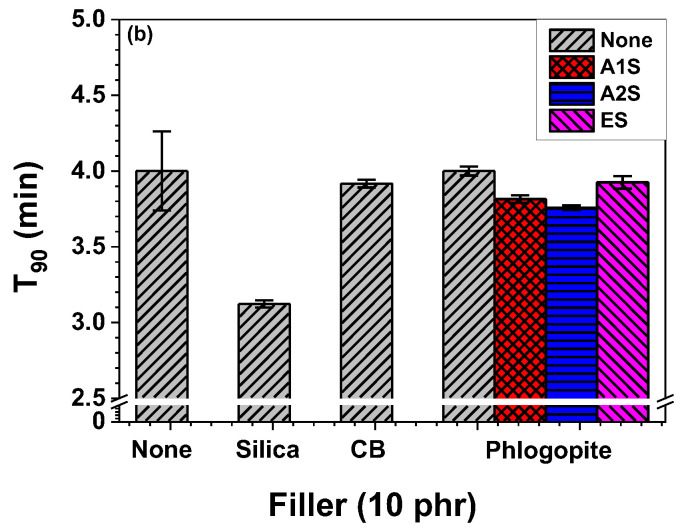
(**a**) Mooney viscosities and (**b**) T_90_ of the pristine NR/EPDM blend and filler-embedded NR/EPDM composites with different compatibilizers.

Viscosities of unvulcanized rubbers are routinely measured using the Mooney viscosity test. Figure 2a shows the Mooney viscosities of the pristine NR/EPDM blend and its filler-incorporated composites, with and without different compatibilizers. The Mooney viscosity was reduced by incorporating inorganic fillers into the NR/EPDM and the Mooney viscosity of the NR/EPDM composite comprising silica was similar to that of the pristine rubber blend. The phlogopite-embedded NR/EPDM rubber composite showed the lowest Mooney viscosity because of the platy architecture of phlogopite. Mica-based inorganic fillers including phlogopite with the platy architecture usually show low Mooney viscosities [46,47]. The different types of silane-based additives barely influenced the Mooney viscosities of the rubber composites. Different fillers and compositions of rubber composites exhibited different curing behaviors. The optimal curing time for rubbers is routinely defined as the time (T_90_) required for the torque to reach 90% of the maximum torque during curing. The incorporation of each filler and the different surface modification hardly influenced the T_90_, as shown in Figure 2b. The surface modifications of phlogopite reduced the curing time of the samples, indicating fast curing. Compared with that of the pristine blend, the T_90_ values decreased with increasing amino moiety concentration, whereas that of the epoxide-functionalized phlogopite-embedded sample barely changed.

**Figure 3:**
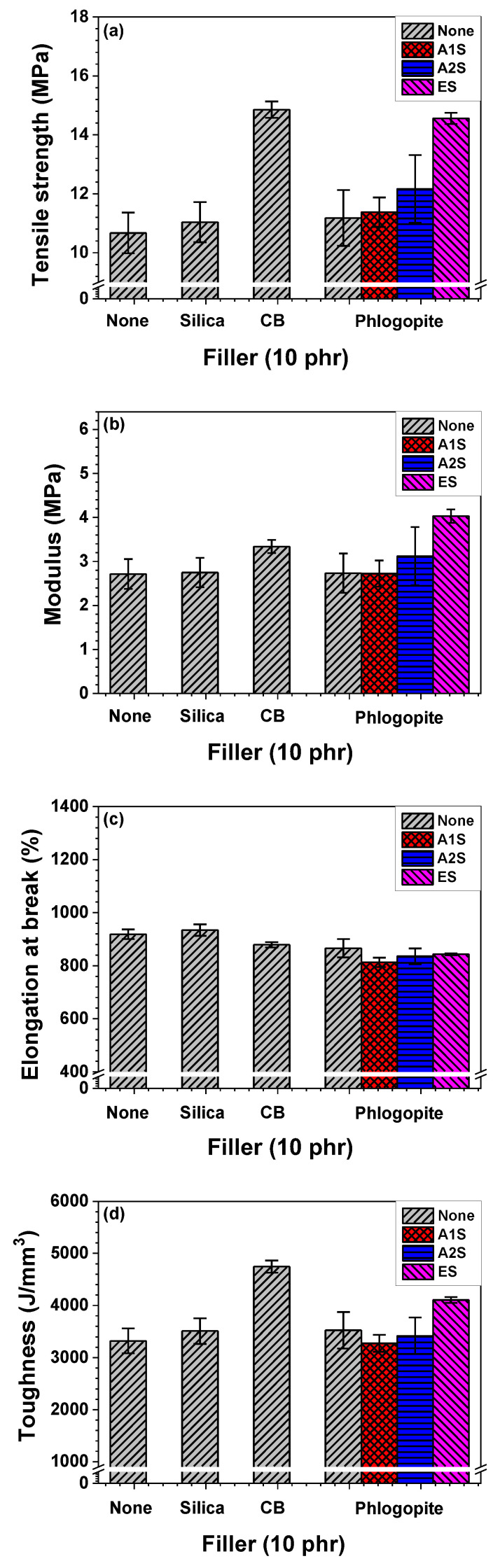

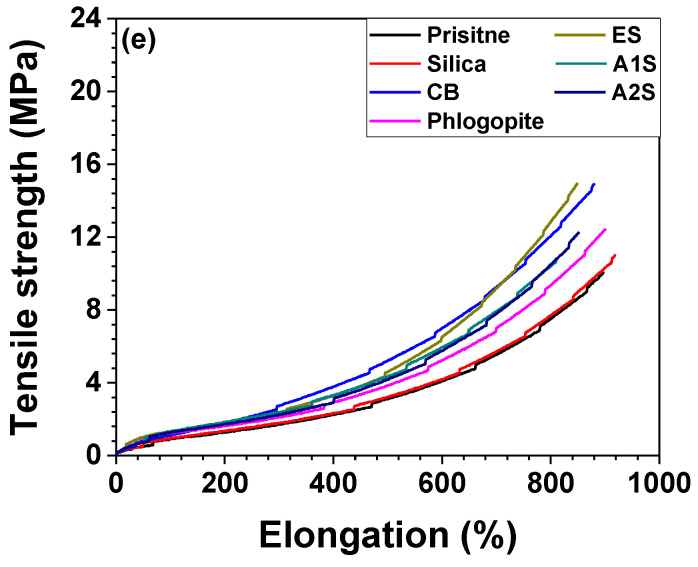
Tensile properties of NR/EPDM blend and composites with different fillers and phlogopite functionalization types (A1S, A2S, and ES): (**a**) Tensile strength, (**b**) modulus, (**c**) elongation at break, and (**d**) toughness, (**e**) stress–strain plots.

Tensile properties of the NR/EPDM blend and composites with different fillers (silica, CB, and phlogopite) and with different phlogopite functionalization types (amino- and epoxide-functionalization) were investigated, as shown in Figure 3. Among the prepared samples, CB-incorporated NR/EPDM composite showed the highest tensile strength (14.9 MPa). The tensile strength of the untreated phlogopite-incorporated NR/EPDM (11.2 MPa) was analogous to that of the silica-embedded NR/EPDM composite (11.0 MPa). However, the functionalization for phlogopite enhanced the tensile strength of the composites. The strength of the amino-functionalized phlogopite-embedded composites increased as a function of their amino group content. Especially, the epoxide-functionalization for phlogopite substantially improved the tensile strength (14.6 MPa) of the NR/EPDM composite consisting of epoxide (ES)-functionalized phlogopite, which was similar to that of CB-infiltrated NR/EPDM composite. The trend of the tensile moduli of the composites was similar to that of their tensile strength. The pristine NR/EPDM blend without filler showed the lowest modulus. The modulus of the phlogopite-incorporated composites was enhanced by phlogopite surface treatment. The modulus of the ES-functionalized phlogopite-embedded composite was superior to that of the CB-embedded composite, indicating that the surface functionalization of phlogopite led to the formation of a physical network between the fillers and rubber matrix. The elongation at break of silica-embedded NR/EPDM composite was analogous to that of the pristine NR/EPDM blend without filler, whereas the elongation at break of the other NR/EPDM composites was reduced by the incorporation of CB or phlogopite into the rubber blend, regardless of functionalization. Although the ES-functionalized phlogopite-reinforced NR/EPDM composite showed the highest modulus, the CB-infiltrated composite showed the highest toughness (energy to break), which was primarily caused by large reduction in elongation at break for phlogopite-incorporated NR/EPDM composites.

**Figure 4:**
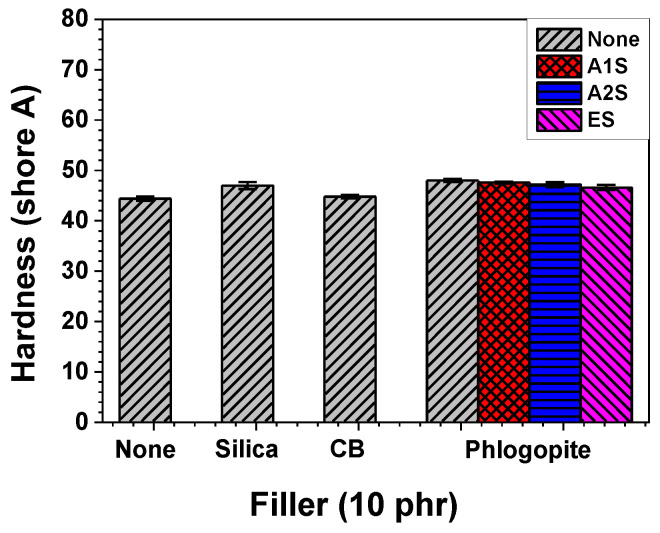
Shore A hardness of the NR/EPDM blend and composites with different fillers and functionalization types (amino- and epoxide-functionalized) for phlogopite.

Resistance can be measured when mechanical indentation or abrasion leads to localized plastic deformation. Hardness is defined as the resistance value obtained from such measurements. The hardness values of the silica- or phlogopite-embedded NR/EPDM composites were slightly higher than those of the pristine NR/EPDM blend, as shown in Figure 4. Unlike their tensile properties, the hardness values of the phlogopite-incorporated composites were barely influenced by the functionalization of the phlogopite surface. These results may be attributed to the low concentration (10 phr) of the fillers.

**Figure 5:**
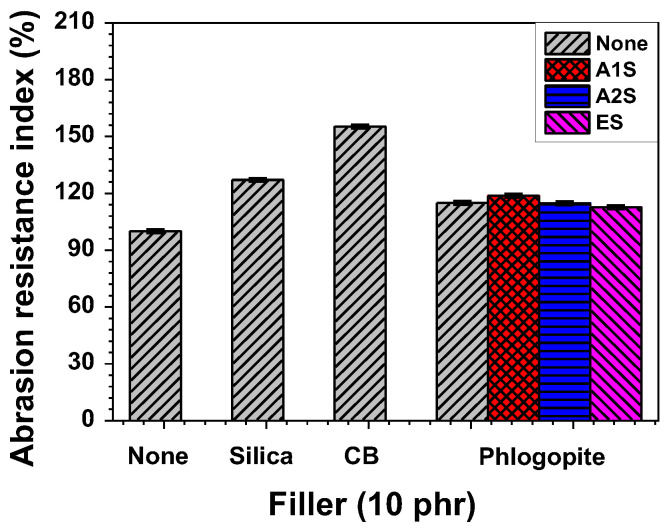
Abrasion resistance index (ARI) of the NR/EPDM blend and composites with different fillers and functionalization types (amino- and epoxide-functionalized) for phlogopite.

Mechanical properties, such as tensile properties and hardness, are shown in Figure 3 and Figure 4. In addition, abrasion resistance index (ARI) was measured to examine the durability toward abrasion, as shown in Figure 5. ARI is a crucial parameter influencing the performance of rubber materials, especially when they are applied to tires, which experience repeated abrasion. The ARI values were calculated using Equation (1). The infiltration of fillers (i.e., silica, CB, and phlogopite) into the NR/EPDM rubber blend increased the ARI of the resultant specimens. In particular, the CB-reinforced composite exhibited the highest ARI value, compared to other filler-embedded rubber composites. The ARI of the rubber composites rarely showed any change, regardless of the phlogopite functionalization.

**Figure 6:**
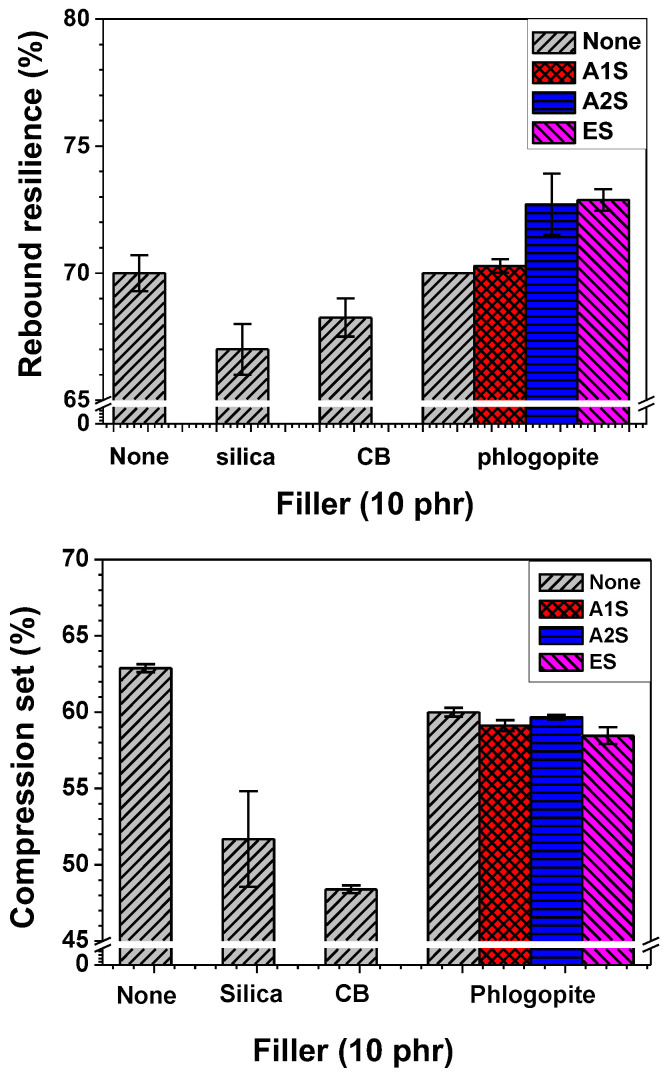
Rebound resilience and compression set of the NR/EPDM blend and composites with different fillers and functionalization types (amino- and epoxide-functionalized) for phlogopite.

The elastic characteristics of rubbers can be investigated by measuring its rebound resilience and compression set, as shown in Figure 6. The rebound resilience refers to a ratio of the energy returned to the energy applied for deformation by an external force. The compression set represents the degree of deformation sustained when the force is removed after a rubber sample is deformed by a force at a high temperature for a certain period of time. Thus, low compression set values indicate high elasticity. The incorporation of A2S- and ES-functionalized phlogopite into the NR/EPDM rubber matrix enhanced the rebound resilience and reduced the compression set, which indicates good elasticity. However, the elasticity of the A1S-functionalized phlogopite/NR/EPDM was similar to that of the untreated phlogopite/NR/EPDM composite owing to the low concentration of amino moieties in A1S, which resulted in low compatibility [48,49,50,51]. The rebound resilience values of the various phlogopite-embedded composites were higher than those of the silica- or CB-incorporated composites, whereas compression set values of phlogopite/NR/EPDM composites were higher than those of silica- or CB-embedded composites. This finding indicates that the elastic properties for the rubber composites containing silica or CB under thermo-pressure conditions during compression set tests were superior to those of phlogopite-incorporated NR/EPDM rubber composites.

**Figure 7:**
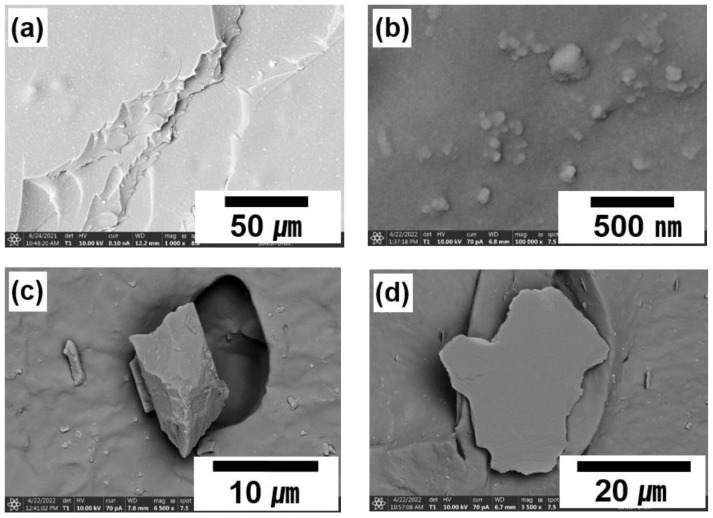

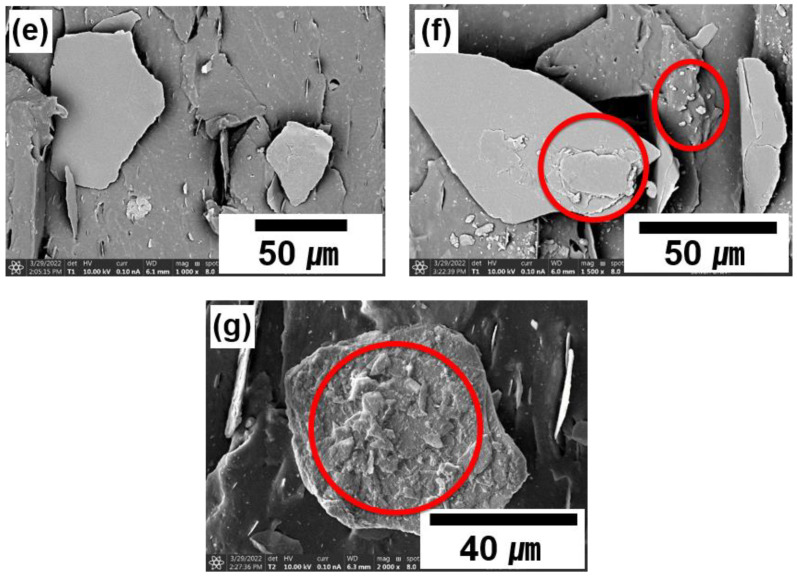
SEM images of fractured NR/EPDM rubber with different fillers (10 phr) and functionalization types (amino- and epoxide-functionalization) for phlogopite: (**a**) None, (**b**) CB, (**c**) silica, (**d**) untreated phlogopite, (**e**) A1S-treated phlogopite, (**f**) A2S-treated phlogopite, and (**g**) ES-treated phlogopite.

**Figure 8:**
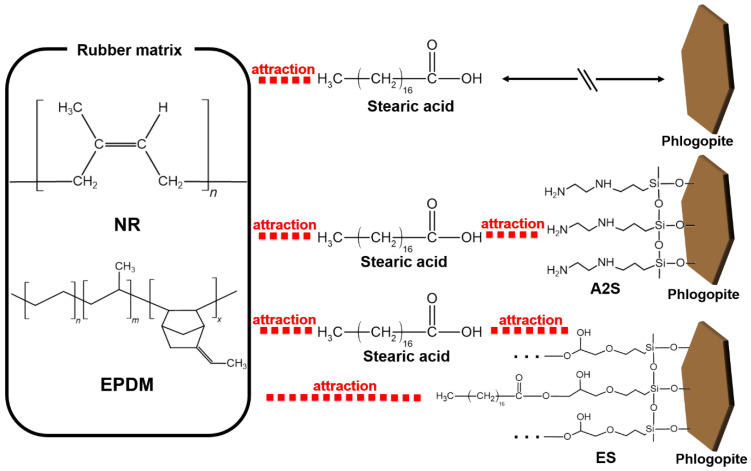
Mechanisms of physical and chemical interactions among rubber matrices, stearic acid, and functionalized phlogopite. *n*, *m*, and *x* indicate the number of each repeating unit.

Figure 7 shows SEM micrographs of fractured NR/EPDM without and with fillers (10 phr). The pristine NR/EPDM blend presented smooth surfaces, as shown in Figure 7a. CB was inhomogeneously dispersed in the NR/EPDM rubber blend, exhibiting agglomeration (Figure 7b) owing to its extremely fine dimensions. The surfaces of untreated and A1S-treated phlogopite were smooth (Figure 7d,e), whereas some parts of the rubber matrix were adsorbed on the surfaces of A2S- and ES-treated phlogopite as shown in Figure 7f,g. The adsorbed rubber on the phlogopite (red circles in Figure 7) indicates excellent compatibility between these phases, thereby increasing the interfacial adhesion and mechanical properties. Figure 8 shows the mechanisms of physical and chemical interactions among the rubber matrix, stearic acid, and functionalized phlogopite. The physical and chemical interactions were caused by A1S/A2S and ES, respectively. The secondary hydroxyl moieties generated during reactions between carboxylic acid and epoxide [52,53,54,55] may contribute additional physical interactions (e.g., hydrogen bonding and dipole–dipole interactions) between hydroxyl and carboxylic groups [56,57].

## 4. Conclusions

The effects of incorporating untreated and treated phlogopite into the NR/EPDM blend were systematically examined in this study. Curing, mechanical, elastic, and morphological properties of the blend and composites were investigated. The properties of the functionalized phlogopite-reinforced NR/EPDM composites were compared with those of rubber composites consisting of typical fillers (i.e., silica and CB) for rubber compounding. The mechanical properties of the untreated phlogopite/NR/EPDM composite were inferior to those of silica- and CB-infiltrated NR/EPDM composites. However, the amino (A1S and A2S) and epoxide-functionalization for phlogopite substantially enhanced the mechanical and elastic properties. Especially, in terms of tensile modulus, the ES-functionalized phlogopite-incorporated composite was the highest. The other properties of the ES-phlogopite/NR/EPDM composite were analogous to those of the CB/NR/EPDM composite. This finding results from good interactions among rubber matrices, stearic acid, and ES-functionalized phlogopite. Thus, the functionalized phlogopite can be utilized in a myriad of rubber applications. Future work should investigate the combined effects of the phlogopite and CB on the properties of NR/EPDM blends.

## Figures and Tables

**Table 1:** Recipes of the rubber compounds.

Rubber Matrix (wt%)							
NR	75	75	75	75	75	75	75
EPDM	25	25	25	25	25	25	25
**Additive (phr)**							
Zinc oxide	5	5	5	5	5	5	5
Stearic acid	2	2	2	2	2	2	2
CB	-	10	-	-	-	-	-
Silica	-	-	10	-	-	-	-
Phlogopite	-	-	-	10	-	-	-
A1S-phlogopite	-	-	-	-	10	-	-
A2S-phlogopite	-	-	-	-	-	10	-
ES-phlogopite	-	-	-	-	-	-	10
TMTD	1	1	1	1	1	1	1
CBS	1	1	1	1	1	1	1
Sulfur	1	1	1	1	1	1	1

## Data Availability

Not applicable.
